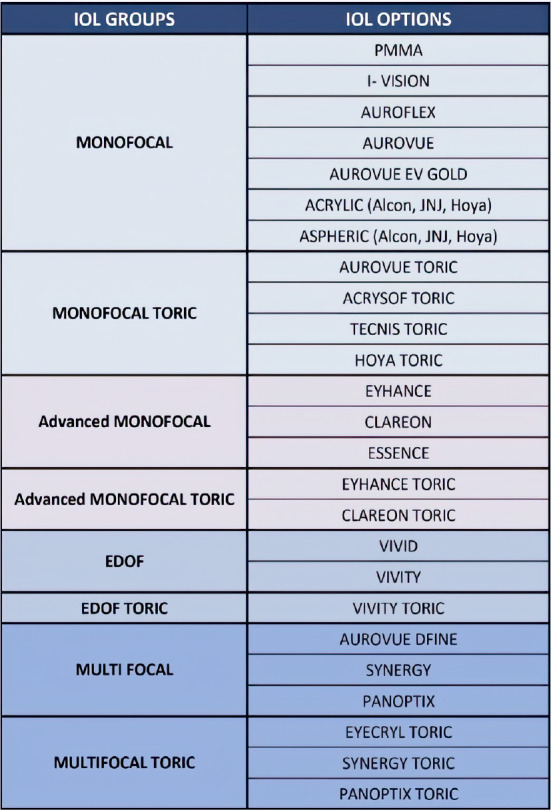# IOLs in India: How and where they are used

**Published:** 2025-09-10

**Authors:** Suganya Anbalagan, Aravind Haripriya, Ravilla D Ravindran

**Affiliations:** 1 Medical Consultant: Cataract and IOL Services, Aravind Eye Hospital, India.; 2 Chief: Cataract and IOL services, Aravind Eye Hospital, India.; 3 Chairman: Aravind Eye Care System, India.

**A wide variety of IOL types are now available in India.**

In India, implanting an intraocular lens (IOL) became standard practice for cataract surgery in the late 1990s. The government supported this by providing grants for patients operated on during outreach eye camps.

According to recent Indian National Blindness & Visual Impairment Survey 2015-2019, majority (94.3%) of cataract operations were performed using intraocular lenses (IOLs). The ease of access to IOLs seem to have improved the cataract surgical coverage (CSC): at best corrected visual acuity (BCVA) levels of <3/60, <6/60 and <6/18, the CSC was 93.2%, 89.0%, and 74.0%, respectively.^1^

In the 1990s, monofocal three piece or single piece polymethylmethacrylate (PMMA) IOL or foldable silicone/acrylic IOLs were the standard IOLs available. Since then, advances in IOL technology and rising patient expectations have led to a wide variety of IOL models, with varying sight outcomes and costs. For patients who can chose, IOL selection is crucial for their visual outcomes and satisfaction.

A wide range of cost effective, locally manufactured IOLs are available in India, alongside more expensive imported IOLs from other countries. Based on the current data, nearly 30% of patients are operated as a result of outreach camps conducted by government or non-governmental organisations.^2^ These patients typically receive MSICS with implantation of single or three-piece PMMA IOLs. These IOLs are locally manufactured, widely available, and and cost around USD 3–4 each.

Walk-in patients at government hospitals usually receive MSICS with a PMMA IOL at no cost. At eye hospitals owned by non-governmental organisations (NGOs), same procedure costs USD 12–50 . In some Indian states, direct walk-in operations for those below the poverty line in government, NGO, or private eye hospitals are reimbursed through the government insurance scheme (Ayushman Bharat). The scheme pays around USD 100 per phacoemulsification operation with a monofocal foldable hydrophobic IOL, or USD 50 for MSICS with a PMMA IOL.

In private hospitals, where patients pay for their own operation, phacoemulsification with a foldable IOL costs around USD 150–250 for locally manufactured monofocal foldable IOLs and more than USD 500 for imported aspheric hydrophobic foldable IOLs. The locally manufactured foldables cost around USD 25–30 while the imported foldables cost USD 80–90.

In our experience, about 85% of patients who have MSICS with rigid PMMA IOL achieve uncorrected Snellen visual acuity (UCVA) of 6/18 or better. Many of them also have reasonable near sight due to some myopic astigmatic error postoperatively. Nearly 25% of these patients have near vision of N8 or better. With PE surgery with foldable IOL, more than 80% of the patients achieve 6/12 or better UCVA for distance, but require spectacle correction for near sight.^3^

Operations using **advanced monofocals** are usually performed for private patients, particularly those with personal health insurance. These IOLs cost around USD 500–600 .

While the locally manufactured **toric IOL** cost around USD 70–80, imported ones cost around USD 200–225 About 1–2% of patients end up requiring re-rotation of the IOL postoperatively to achieve optimal results.^3^ Toric IOLs are implanted in about 12–20% of paying patients at Aravind.^4^

**Multifocal and extended depth-of-focus (EDOF) IOLs** cost around USD 500–600. At Aravind, the uptake of multifocal or EDOF IOL surgery is around 5% of paying patients.

**Table 1:** Intraocular lens (IOL) types commonly used at Aravind Eye Care System